# Short- and Long-Term Effectiveness of Brief Intensive Trauma Treatment for Adolescents With Posttraumatic Stress Disorder and Their Caregivers: Protocol for a Multicenter Randomized Controlled Trial

**DOI:** 10.2196/66115

**Published:** 2025-04-30

**Authors:** Myrna M Westerveld, Malindi van der Mheen, Rik Knipschild, Kim Maijer, Marieke E de Keizer-Altink, Nina Albisser, Marielle J E Hoekstra, Anne Timmermans-Jansen, Rosa Zijp, Anne A Krabbendam, Francisca J A (Bonny) van Steensel, Kees-Jan Kan, Chaim Huyser, Wouter G Staal, Elisabeth M W J Utens, Ramón J L Lindauer

**Affiliations:** 1 Department of Child and Adolescent Psychiatry Amsterdam University Medical Center Location University of Amsterdam Amsterdam The Netherlands; 2 Academic Center for Child and Adolescent Psychiatry Levvel Amsterdam The Netherlands; 3 Amsterdam Public Health Research Institute Amsterdam University Medical Center Amsterdam The Netherlands; 4 Child and Adolescent Psychiatry Karakter Almelo The Netherlands; 5 Forensic Care Specialists De Waag Amsterdam The Netherlands; 6 Child and Adolescent Psychiatry Mental Health Caribbean Kralendijk Netherlands Antilles; 7 Department of Child and Adolescent Psychiatry Leiden University Medical Center, Curium Leiden The Netherlands; 8 Leiden University of Applied Sciences Leiden The Netherlands; 9 Research Institute of Child Development and Education University of Amsterdam Amsterdam The Netherlands; 10 Department of Psychiatry Child and Adolescent Psychiatry Nijmegen The Netherlands; 11 Department of Psychiatry Radboud University Medical Centre Nijmegen The Netherlands; 12 Leiden Institution for Brain and Cognition Leiden The Netherlands

**Keywords:** brief intensive trauma treatment, adolescents, stress disorder, post-traumatic, adverse childhood experiences, eye movement desensitization reprocessing, trauma-focused cognitive behavioral therapy, Caribbean, randomized controlled trials, multicenter studies

## Abstract

**Background:**

Childhood trauma is pervasive, with approximately 50% of adolescents experiencing at least one potentially traumatic event before adulthood. Eight percent to 33% of potentially traumatic event–exposed adolescents develop posttraumatic stress disorder (PTSD), which can cause extreme suffering and coincides with numerous comorbid illnesses and high-risk behaviors. PTSD can be effectively treated in adolescents through weekly sessions of eye movement desensitization and reprocessing or trauma-focused cognitive behavioral therapy. Despite the availability of these treatments, numerous severely traumatized adolescents do not receive available treatment options due to high treatment avoidance. In adolescents who receive care, a large group of youth does not experience a sufficient symptom decrease after regular treatment. In addition, dropout rates during prolonged treatment are substantial, varying between 10% and 30%. This underscores the need for innovative and brief trauma treatment. Pilot studies indicate that Brief Intensive Trauma Treatment (BITT) can be a safe and effective treatment for adolescents with PTSD. However, randomized controlled trials on its effectiveness are crucial and urgently needed.

**Objective:**

This is the first study to test the effectiveness of a 1-week BITT in adolescents with PTSD and comorbid symptoms and their caregivers.

**Methods:**

This multicenter, single-blinded randomized controlled trial will be conducted in 4 youth care centers in the European and Caribbean Netherlands: Levvel, Karakter, Fornhese-GGz Centraal, and Mental Health Caribbean (Bonaire). We will randomize adolescents (12-18 years old) with PTSD to a BITT (n=50) or waitlist control group (WLCG; n=50). BITT comprises 1-week (ie, 5 consecutive workdays) intensive trauma treatment, encompassing daily 90-minute manualized sessions of trauma-focused cognitive behavioral therapy and eye movement desensitization and reprocessing. The day begins and ends with psychomotor therapy. Caregivers receive daily parental counseling sessions consisting of psychoeducation and social support skill training. We will conduct measurements at similar intervals for both groups: at baseline; directly after BITT or WLCG; and at 3, 6, and 9 months’ follow-up. The WLCG will receive BITT after the 3-month follow-up assessment. We will assess all study parameters using digital or face-to-face questionnaires and semistructured interviews. We will assess the primary outcome PTSD symptoms using the Child and Adolescent Trauma Screen 2 (CATS-2) and the Clinician-Administered PTSD Scale for *DSM-5* (*Diagnostic and Statistical Manual of Mental Disorders* [Fifth Edition])-Child/Adolescent Version (CAPS-CA-5).

**Results:**

As of September 2022, we enrolled 104 participants. Data will be collected until December 2025. Results are expected to be published in the summer of 2026.

**Conclusions:**

This first, innovative study on BITT’s effectiveness may enhance treatment outcomes for PTSD by preventing dropout, reducing avoidance, shortening therapy duration, and empowering therapists by working together intensively. This research will provide valuable insights across cultures for treating severely traumatized adolescents who do not benefit sufficiently from regular treatment.

**Trial Registration:**

ClinicalTrials.gov NCT06143982, http://clinicaltrials.gov/ct2/show/NCT06143982

**International Registered Report Identifier (IRRID):**

DERR1-10.2196/66115

## Introduction

Childhood trauma is pervasive, with around 50% of adolescents experiencing at least one potentially traumatic event (PTE) before adulthood [1-3]. Posttraumatic stress disorder (PTSD) is the most common disorder that can develop after experiencing PTE and occurs in around 16% of PTE-exposed adolescents, with a range of 8%-33%. Importantly, the variability within this range is influenced by the type of trauma and gender. In particular, PTSD is less prevalent in boys exposed to noninterpersonal trauma, while girls exposed to interpersonal trauma are at a greater risk of developing PTSD [4]. PTSD manifests through symptoms such as re-experiencing (ie, flashbacks, nightmares, dissociation, repetitive distressing images or sensations, and physical sensations such as pain, sweating, nausea, or trembling), avoidance and emotional numbing, negative alterations in cognitions and mood, and hyperarousal (ie, irritability, angry outbursts, sleep disturbances, and difficulty concentrating) [5].

Furthermore, PTSD significantly affects an individual’s quality of life [6,7]. Adolescents with PTSD have been found to show high rates of psychopathology at the age of 18 years, including 48.8% engaging in self-harm, 20.1% attempting suicide, 54.7% diagnosed with major depressive disorder, 23.8% with generalized anxiety disorder, and 27% with conduct disorder [8]. In addition, subclinical PTSD (sPTSD) in adolescents is often neglected. However, adolescents with sPTSD can experience similar distress as adolescents with full-blown PTSD [9]. These findings emphasize the critical importance of early treatment for both clinical and sPTSD.

The internationally recommended treatment guidelines for PTSD are weekly sessions of eye movement desensitization and reprocessing (EMDR) or trauma-focused cognitive behavior therapy (TF-CBT) [10-13]. However, 30% of individuals do not benefit enough from evidence-based PTSD treatments. This might be explained by PTSD symptom severity and high rates of psychiatric comorbidity within this group of patients. Notably, greater symptom severity and high rates of comorbidity are associated with reduced treatment success [14]. In addition, dropout rates during prolonged treatment are substantial, varying between 10% and 30% [15-18]. The duration of therapy and treatment avoidance potentially cause these dropout rates. Current treatment strategies are prolonged (at least 3-4 months) and, therefore, subject to engagement and compliance barriers. For example, adolescents can be reluctant to engage in therapy due to fear of re-experiencing their trauma over a long period of time [19]. Due to frequent appointments over a longer period, adolescents miss school time and the opportunities to engage in hobbies, social activities, and peer contact, hampering healthy development and resulting in therapy disengagement. Notably, a greater number of therapy sessions is found to be an important predictor of dropout in adolescents with trauma [20]. These findings underline that innovative trauma treatments are crucial and that treatments in a novel, brief, and intensive format are urgent.

Brief Intensive Trauma Treatments (BITT) entail multiple trauma-focused sessions, often integrating various treatment modalities simultaneously. Recent studies have indicated that BITT for adults can serve as a safe and effective strategy for reducing PTSD [21-24]. These studies have yielded promising outcomes in adults, characterized by notably low dropout rates and minimal adverse events. Unfortunately, evidence of the effectiveness of brief trauma treatments in children and adolescents is limited. To date, only 9 studies have shown promising results [25-33]. However, 8 of the 9 studies were conducted without control groups and all studies had relatively small sample sizes (ie, N=10, N=27, N=22, N=15, N=15, N=33, N=44, N=27, and N=36). Hence, from the perspective of clinical relevance and optimizing treatment and healthy development of children with trauma, randomized controlled trials (RCTs) investigating the effectiveness of BITT in adolescents are urgently needed. This was the rationale for the present innovative multicenter RCT. This RCT follows up on a pilot study (N=22) of the same BITT program, which previously demonstrated promising results, including a reduction in PTSD symptoms up to 3 months post treatment [34].

## Methods

### Aims

#### Primary Objective

The primary objective of this RCT is to assess the effectiveness of a BITT compared to a waitlist control group (WLCG) in reducing symptoms of PTSD among adolescents aged 12-18 years.

#### Secondary Objectives

The secondary objectives of the study are to investigate whether BITT for adolescents with PTSD:

1. in comparison to a WLCG, demonstrates effectiveness in reducing comorbid psychiatric symptoms (ie, anxiety, depression, and anger) and improving the quality of life;
2. is safe (ie, does not increase suicide attempts or self-harm)
3. reduces dropout rates;
4. is affected by moderators (ie, age, gender, socioeconomic status [SES], type of trauma, number of traumatic events, comorbidity, caregivers PTSD, and treatment center) in its effect on PTSD symptoms and BITT dropout (moderators: residency [ie, urban and rural area] and ethnicity).

### Study Design

**Figure 1:**
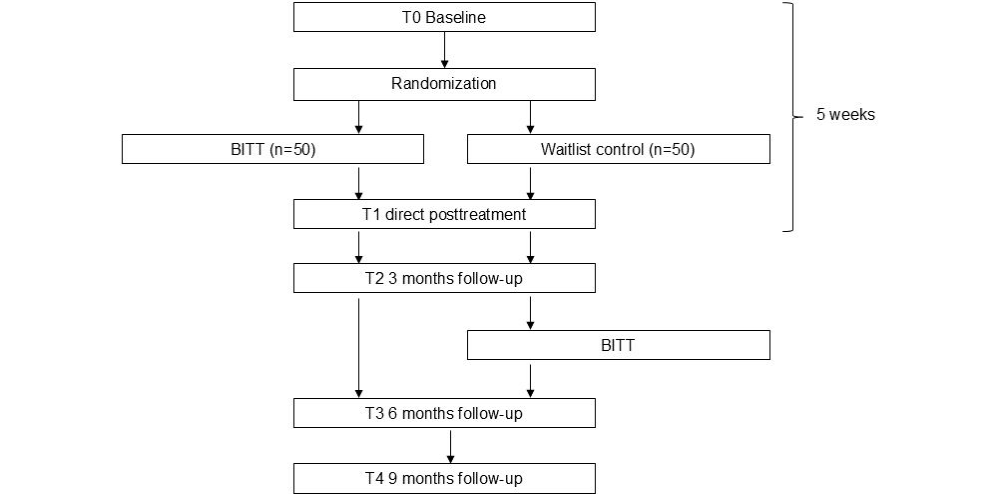
Flowchart of the randomized controlled trial design. BITT: Brief Intensive Trauma Treatment.

In this multicenter, single-blinded RCT, we will examine the effectiveness of BITT compared to a WLCG. Participants will be recruited over 27 months (October 2022-December 2024). To determine the effectiveness of BITT, we will compare the outcomes of the BITT intervention group (n=50) with the outcomes of a WLCG (n=50). We will conduct assessments at similar time points for both groups: at baseline (T0), directly after BITT or WLCG (T1), and at 3 (T2), 6 (T3), and 9 (T4) months follow-up after the baseline measurement. The WLCG will receive BITT after the 3-month follow-up assessment. Figure 1 provides an overview of the study design.

The treatment phase of BITT lasts only 1 week (ie, 5 consecutive workdays). The WLCG will not receive psychological care (ie, EMDR and TF-CBT) during this week. After this week, participants from the BITT group will follow regular follow-up care, which usually consists of EMDR and TF-CBT. Apart from BITT, the WLCG can receive any treatment including EMDR and TF-CBT, during this period. We will monitor and register all provided care in the research database (inclusion and exclusion criteria listed in Textbox 1).

**Inclusion and exclusion criteria.**

Inclusion criteria

Participants must meet all of the following criteria:

- 12-18 years of age;
- A history of psychological trauma (as measured by the Life Events Checklist of the Clinician-Administered Posttraumatic Stress Disorder Scale for Children and Adolescents in the *DSM-5 [Diagnostic and Statistical Manual of Mental Disorders* (Fifth Edition)]) [35,36];
- At least subthreshold posttraumatic stress disorder criteria, conform to the Clinician-Administered PTSD Scale for Children and Adolescents *DSM-5*, that is:
  - fully meets criteria A, F, and G, and at least one symptom of criteria B, C, D, and E;
  - fully meets criteria A, F, G, and at least one of the B, C, D, or E symptom clusters;
- The adolescent must provide written informed consent. For adolescents aged 12-15 years, all legal guardians must also provide consent.

Exclusion criteria

Adolescents who meet any of the following criteria will be excluded from participation in this study:

- inability to speak and write Dutch;
- estimated or determined intellectual disability (IQ <70);
- having ongoing trauma by a parent who is part of the adolescent’s current primary care system.

### Power Analyses

A power analysis in R (R Core Team) [37] (script available on request) showed that a sample size of n=66 (33 in the direct group and 33 in the WLCG) is sufficient to reach a required power of at least 80% when assuming Cohen *d*=0.5. Regarding trauma-focused therapies, we expected a dropout rate of around 25% [15-17], implying that we would need at least 88 (ie, 66 divided by 0.75) participants. To ensure sufficient statistical power, we aimed for a total sample size of 100. This sample size will also be sufficient when adding the covariates to the model.

### Participant Recruitment and Procedure

We will conduct this study in four youth care centers in the European and Caribbean Netherlands: Levvel, Karakter, Fornhese-GGz Centraal, and Mental Health Caribbean (Bonaire). Centers were eligible to participate if they had extensive experience in treating chronically traumatized children and adolescents, were trained in EMDR and TF-CBT, had experienced systemic therapists and psychomotor therapists, and had the capacity to implement the study. The therapist from the participating center will verbally inform eligible adolescents about the study and provide them with an information letter. In addition, the adolescent and their caregivers will be offered the opportunity to discuss the study with the researcher to receive more information about the study and ask questions. The researcher will discuss all referrals with an experienced child and adolescent psychiatrist with expertise in PTSD. All adolescents will provide written informed consent, and legal caregivers of adolescents aged 12-15 years will also provide consent.

### Randomization

After obtaining written informed consent from participants, an independent researcher will randomize them to either the BITT group or WLCG using computerized block-wise randomization in Castor Electronic Data Capture [38]. We will use random block sizes to avoid predictability for the researchers and we will stratify by center. The researcher completing the assessments will be blinded to the group allocation (ie, BITT vs WLC). We will explicitly instruct participants not to communicate their group allocation to the researcher.

### Treatment

#### Pretreatment

A personalized “crisis prevention plan” will be developed individually with all participants and, if applicable, their caregivers before the treatment phase. The crisis prevention plan will include the following:

1. Possible risk factors to consider (eg, self-harm, suicidality, alcohol and drug abuse, and aggression);
2. Signs and tools for maintaining stability and preventing deterioration;
3. Clear arrangements regarding whom to contact in case of emergency (eg, the main responsible clinician during working hours and local crisis service outside regular working hours) with up-to-date contact information.

Furthermore, before starting BITT, a personalized treatment plan will be developed with specific trauma targets in close collaboration with the adolescent, caregivers, and BITT therapist. The therapist will also be assigned as a personal case manager for individual adolescents throughout the BITT week. The BITT therapist will provide psychoeducation concerning PTSD symptoms and intensive treatment to adolescents and caregivers before the start of BITT.

#### The BITT

BITT consists of a 1-week (ie, 5 consecutive workdays) intensive trauma treatment with 2 daily manualized therapy sessions comprising TF-CBT (90 min) in the morning followed by EMDR (90 min) in the afternoon (ie, 10 sessions in total; 5 TF-CBT and 5 EMDR). The 90-minute session duration is based on the results of a previous pilot study into BITT for adolescents, which indicated that the burden of 90-minute sessions was evaluated as acceptable [25]. Both TF-CBT and EMDR therapy are effective and well-established treatments for children and adolescents suffering from trauma [15,39]. In both TF-CBT and EMDR, the primary objective is to address and reprocess traumatic memories and diminish the distressing emotions associated with traumatic life events. In TF-CBT, therapists apply gradual exposure from the beginning by having adolescents create a trauma narrative. Gradual exposure is applied by first telling the trauma narrative aloud, then writing it, and then reading it aloud. This also helps adolescents modify dysfunctional trauma-associated cognitions. On the last day of the BITT, the adolescent shares the trauma narrative with the caregivers during a so-called social sharing, which is a module of TF-CBT. Often this is the first time caregivers and adolescents engage in a conversation about the traumatic experience. The caregiver or caregivers are then provided with the opportunity to acknowledge the harm experienced by the adolescent, reflect on their role as a parent, and consider how they could have acted differently. This interaction is seen as an important step in trauma processing. In EMDR, the adolescent recalls the traumatic memories while simultaneously making horizontal eye movements and performing tasks with a high working memory load [40]. Therapists will use the Dutch EMDR protocol for children and adolescents [41,42].

The sequence of TF-CBT first and EMDR second is related to a proven better treatment outcome compared to the reversed sequence [43]. Experienced therapists trained in EMDR and TF-CBT will conduct all treatment sessions. Different therapists will provide sessions that conform to a so-called “therapist rotation” scheme with a maximum of 3-4 therapists per adolescent per week. Therapist rotation is applied since it seems to decrease avoidance in adolescents to engage in trauma therapy [44]. Treatment will take place in an outpatient setting.

Between therapy sessions, adolescents will participate in psychomotor therapy (PMT). PMT is a therapeutic approach in which physical movement and bodily awareness play a central role. In the morning of every BITT day, the focus during PMT will be on bodily awareness (eg, observing body signals related to PTSD) and in the afternoon, the focus will be on relaxation. Existing evidence shows that body- and movement-oriented interventions have a positive effect on diminishing PTSD symptoms [45,46].

During parental counseling, primary caregivers will receive psychoeducation, social support skill training, and social sharing (a total of 5 sessions of 90 min). Research has shown that involving caregivers is a crucial element in the treatment of children and adolescents, which contributes to fewer dropouts and increased treatment effects [39].

Adolescents will start and end the day together. PMT sessions are provided in a group setting. The TF-CBT and EMDR sessions are provided individually. Table 1 provides an overview of the therapy program.

**Table 1:** Therapy program.

Time	BITT week				
	Monday	Tuesday	Wednesday	Thursday	Friday
9-9:15 AM	Start (8:45 AM)	Start	Start	Start	Start
9:15-10:15 AM	PMT^a^	PMT	PMT	PMT	PMT
10:30 AM-12 PM	TF-CBT^b^ Parental counseling^c^	TF-CBT	TF-CBT Parental counseling	TF-CBT	EMDR
12-1 PM	Break	Break	Break	Break	Break
1:15-2:45 PM	EMDR^d^	EMDR Parental counseling	EMDR	EMDR Parental counseling	Social sharing
3-3:45 PM	PMT	PMT	PMT	PMT	Closing (3-3:30 PM)
4-4:15 PM	Closing	Closing	Closing	Closing	N/A^e^

^a^ PMT: psychomotor therapy.

^b^TF-CBT: trauma-focused cognitive behavioral therapy.

^c^Caregivers receive parental counselling at the same time that the adolescent receives trauma therapy.

^d^EMDR: eye movement desensitization and reprocessing.

^e^N/A: not applicable.

### Training and Implementation

All health care professionals involved in BITT are trained by expert therapists before providing treatment, ensuring the quality of care. During this 1-day training, all modules of the BITT will be taught. In addition, the TF-CBT therapists will follow a 1-day training about the BITT-specific TF-CBT elements. Furthermore, BITT therapists from all participating centers without a TF-CBT or EMDR license will be trained by expert and licensed senior TF-CBT and EMDR supervisors from Levvel. These expert therapists were also involved in the development of the BITT program and have nationally renowned expertise. The researcher determining the PTSD symptoms during the study will be trained in the Clinician-Administered PTSD Scale for *DSM-5* (*Diagnostic and Statistical Manual of Mental Disorders* [Fifth Edition])-Child/Adolescent Version (CAPS-CA-5). During the BITT, the supervisors from Levvel will provide daily supervision to all therapists at the participating centers during a daily multidisciplinary consultation. During this consultation, all BITT therapists work closely together enhancing a clear focus on the treatment plan.

### Treatment Integrity

During the BITT, 2 supervisors from Levvel will provide daily supervision to all therapists of the participating centers in close collaboration with dedicated experts from those centers. In this way, the consistency and quality of the BITT are monitored across sites. Furthermore, therapists will complete detailed therapy checklists that will be assessed by a researcher to ensure that the treatments are delivered as intended by the protocol. In addition, therapists will record the therapy sessions with audiotapes if the participant provides permission. A total of 2 trained psychologists will independently code a random 15% of these therapy sessions to assess the treatment integrity.

### Assessment Instruments

The assessments of the primary and secondary outcomes and demographic predictor variables consist of digital questionnaires and digital or face-to-face semistructured interviews administered to both the adolescents and one of their caregivers. The measurement instrument we selected for assessing the primary outcome, namely the CAPS-CA-5, is considered the golden standard for measuring PTSD. In selecting the questionnaires, we aimed to select short surveys with adequate psychometric properties. If the participant provides permission, we will audiotape the semistructured interviews. A total of 2 trained psychologists will independently code a random 15% of these sessions to measure the interrater reliability. All questionnaires will be completed in Castor Electronic Data Capture [38]. The completion time of the assessments for adolescents varies from 75 to 120 minutes per assessment moment and for the caregiver from 30 to 65 minutes per assessment moment. The overall time for therapists to complete the assessments is 20 minutes. Table 2 provides an overview of the study parameters.

**Table 2:** Study parameters.

Outcomes		Assessment instrument	T0^a^	BITT^b^	T1^c^	T2^d^	T3^e^	T4^f^
Demographics		—^g^	A^h^, C^i^	—	—	—	—	—
**Primary**								
	PTSD^j^ symptoms	CATS-2^k^	A, C	A	A, C	A, C	A, C	A, C
	Clinical diagnosis of PTSD	CAPS-CA-5^l^	A	—	A	A	A	A
**Secondary**								
	Depression symptoms	PROMIS^m^ depression	A	A	A	A	A	A
	Anxiety symptoms	PROMIS anxiety	A	A	A	A	A	A
	Anger symptoms	PROMIS anger	A	A	A	A	A	A
	Quality of life	EQ-5D-Y-3L	A, C	A	A, C	A, C	A, C	A, C
	Risk-behavior and safety	Risk behavior and safety	A	—	A	A	A	A
	Parental PTSD	PCL-5^n^ including LEC-5^o^	C	—	C	C	C	C
	Rating of sessions and therapy	ORS^p^ and SRS^q^	—	A	—	—	—	—
**Qualitative**								
	BITT^r^ experience	Qualitative interview	—	—	A, C, T^s^	A, C	—	—

^a^T0: baseline.

^b^During the BITT questionnaires are assessed daily.

^c^T1: directly after BITT or WLCG.

^d^T2: 3 months follow-up.

^e^T3: 6 months follow-up.

^f^T4: 9 months follow-up.

^g^Not applicable.

^h^A: adolescent.

^i^C: caregiver.

^j^PTSD: posttraumatic stress disorder.

^k^CATS-2: Child and Adolescent Trauma Screen.

^l^CAPS-CA-5: Clinician-Administered PTSD Scale for *DSM-5 (Diagnostic and Statistical Manual of Mental Disorders* [Fifth Edition])-Child/Adolescent Version.

^m^PROMIS: Patient-Reported Outcomes Measurement Information System.

^n^PCL-5: PTSD Checklist for *DSM-5*.

^o^LEC-5: Life Events Checklist for *DSM-5*.

^p^ORS: Child Outcome Rating Scale.

^q^SRS: Session Rating Scale.

^r^BITT: Brief Intensive Trauma Treatment.

^s^T: therapist.

### Primary Outcomes

#### Child and Adolescent Trauma Screen

The Dutch version of the Child and Adolescent Trauma Screen 2 (CATS-2) [47,48] is a self-report and proxy-report measure for children (aged 7-18 years) and their caregivers. The questionnaire contains a list of potentially traumatic life events (16 items) and questions about PTSD symptoms (25 items; ie, re-experiencing, avoidance, emotional numbing, negative alterations in cognitions and mood, and hyperarousal). Respondents can answer each potentially traumatic life event with yes (scored as 1) or no (scored as 0). The questions about PTSD symptoms are rated on a 4-point Likert scale (range 0-3). A score of ≥2 indicates positive screening for PTSD symptoms. The highest possible total score is 60, with higher scores reflecting a higher severity of symptoms. The CATS-2 also asks informants to indicate whether the symptoms cause difficulties in daily life (yes or no). The CATS-2 has adequate psychometric properties [48].

#### The CAPS-CA-5

The CAPS-CA-5 [35,36,49] is used to determine the presence of PTSD based on the *DSM-5* criteria. In addition, with the CAPS-CA-5, we will collect information about the number of traumatic events and the type of experienced trauma. The CAPS-CA-5 is a 30-item structured clinical interview suitable for children aged 8-18 years. In addition, the CAPS-CA includes a Life Events Checklist consisting of 26 items related to stressful life events. The interviewer assesses per event if the adolescent has “experienced it,” “witnessed it,” “heard about it,” “is not sure about it,” or “never experienced it.”

Afterward, the interviewer assesses the frequency and intensity of PTSD symptoms experienced in the past month using a 5-point Likert scale (range 0-4). A score of ≥2 indicates the presence of a symptom. PTSD is present when an A criterion event has occurred, when symptoms are present for more than 1 month (criterion F), when the symptoms affect the adolescent’s daily life (criterion G), and when there is at least one symptom of re-experiencing (criterion B) and avoidance (criterion C) and 2 symptoms of emotional numbing, negative alterations in cognitions and mood (criterion D), and hyperarousal (criterion E). Subthreshold PTSD is present when the adolescent fully meets criteria A, F, and G and has at least one symptom of criteria B, C, D, and E, or has at least one of the B, C, D, or E symptom clusters. The highest possible total score is 80, with higher scores reflecting a greater severity of symptoms. The Dutch version of CAPS-CA-5 has adequate psychometric properties [35,49].

### Secondary Outcomes

#### Patient-Reported Outcomes Measurement Information System

The Patient-Reported Outcomes Measurement Information System (PROMIS) [50] is a questionnaire for children aged 8-17 years and will be used to measure anxiety (8 items), depression (8 items), and anger symptoms (9 items). The questions are rated on a 5-point Likert scale (range 0-4). The highest possible total score for anxiety and depression is 32 and 36 for anger, with higher scores reflecting a higher severity of symptoms. Raw scores will be converted to *t* scores. The PROMIS anger, anxiety, and depression scales displayed sufficient psychometric properties within the Dutch population [51,52].

#### The EQ-5D-Y-3L

The EQ-5D-Y-3L [53] assesses the perceived quality of life in children aged 8-15 years. This questionnaire maps the quality of life based on 5 dimensions: mobility, self-care, usual activities, pain, and anxiety or depression. Both self-report and caregiver proxy versions will be used. All 5 items of the EQ-5D-Y-3L are rated on a 3-point scale, indicating levels of difficulty as “no problems,” “some problems,” or “a lot of problems.” The highest possible total score is 15, with higher scores reflecting a higher severity of symptoms. The EQ-5D-Y-3L has adequate psychometric properties [54].

#### Risk-Behavior and Safety Questionnaire

We will assess risk behaviors by administering a self-report questionnaire based on previous work by Hendriks et al [25], who administered this questionnaire to children aged 12-18 years. This questionnaire assesses suicidal ideation, self-harm, and aggressive behavior with 8 items using an 11-point Likert scale, ranging from “no, not at all” (=0) to “yes, very much” (=10) [25]. Higher scores reflect a higher severity of symptoms. Alcohol use, drug use, inclination to medication, crisis contacts with mental health care professionals, and psychiatric hospitalization will be measured with 7 items on a dichotomous scale (yes or no). The psychometric properties of this instrument have not yet been examined.

#### PTSD Checklist for DSM-5

We will evaluate the presence and severity of the caregivers’ posttraumatic stress symptoms with the Dutch version of the PTSD Checklist for *DSM-5* (PCL-5) [55] The PCL-5 is a 20-item self-report questionnaire gauging the 4 cluster symptoms of PTSD. The caregiver indicates the degree of burden caused by each symptom on a 5-point Likert scale ranging from 0 (“not at all”) to 4 (“extremely”). The severity of symptoms can be determined either by adding the scores of the items within each of the four clusters or by summing all 20 items. The highest possible total score is 80, with higher scores reflecting a higher severity of symptoms. The PCL-5 has adequate psychometric properties [56].

#### Life Events Checklist for DSM-5

We will administer the Dutch version of the Life Events Checklist for *DSM-5* (LEC-5) [57] to assess the caregivers’ exposure to trauma. This is a self-report questionnaire that consists of 17 items related to stressful life events. The caregiver will specify their experience of each event by selecting from the options “happened to me,” “witnessed it,” “learned about it through an affected friend or family member,” “exposed through work,” or “unsure or not applicable.” The LEC-5 has adequate psychometric properties [58].

#### Outcome Rating Scale and Session Rating Scale

We will assess the adolescents' well-being after each BITT day through the Dutch version of the Child Outcome Rating Scale (ORS) [59]. Adolescents will mark their well-being on 4 visual analog scales that range from 0 to 10, providing information on their well-being (“How am I doing?”), well-being at home (“How are things at home?”), school (“How are things at school?”), and their overall sense of well-being (“How is everything going?”).

We will use the Child Session Rating Scale (SRS) [60] to assess how the child experienced the BITT sessions. Respondents will be asked to rate on a visual analog scale ranging from 0 to 10 the extent to which they felt that “they were listened to,” “what was done and talked about was important to them,” “they liked what was done,” and “hoped they would do something similar next time.”

Both the ORS and SRS have adequate psychometric properties in an adult population [61].

#### Demographics

We will collect demographic information by asking the adolescents and one of the caregivers at baseline about: age, gender, educational level, SES, ethnicity, residency (ie, urban and rural area), and previous and current psychological or psychiatric treatment (eg, psychotherapy, trauma therapy, and medication).

### Qualitative Secondary Study Parameters: Qualitative Interview About BITT Experiences

We will conduct interviews with adolescents, caregivers, and therapists about their experience with BITT in collaboration with 2 of our experts by experience (bachelor education level). Together with the 2 experts by experience, we will develop a broad topic list about experienced benefits and possible improvements of BITT. Topics will be related to adolescents’, caregivers’, and therapists’ preparation for and expectations of BITT, the effects of BITT, the added value of BITT compared to regular trauma therapy, and follow-up treatment. These interviews will be conducted individually with around 15 adolescents, caregivers, and therapists. In addition, we will hold focus groups with the therapists to collect their perspectives regarding BITT. We will conduct interviews 1 month after BITT. The 2 experts by experience will be closely involved in the preparation, conduction, assessment, and analyses of the qualitative interviews.

### Statistical Analysis

We will conduct the analyses in consultation with a statistical expert. Data analyses will include the following:

1. We will define the effects of BITT in terms of group differences between the BITT and WLCG. These differences will be tested according to both the intention-to-treat-principle and completers’ analyses. That is, the pre, post, and follow-up changes (BITT vs WLCG) on the continuous outcomes (ie, trauma symptoms, anxiety symptoms, depression symptoms, anger symptoms, quality of life, and self-harm) will be tested with mixed models; dichotomous outcomes (ie, suicide attempts and dropout) with generalized estimating equation (GEE).
2. Mixed models will also be used to investigate possible moderation effects, which concern effects of (1) age, gender, SES, type of trauma, number of traumatic events, comorbidity, caregivers’ PTSD, and treatment center on PTSD; and (2) residency (ie, urban and rural) and ethnicity on BITT dropout.
3. The qualitative interviews about the experiences of adolescents, caregivers, and therapists with regard to BITT will be verbatim transcribed and analyzed to conform to qualitative research standards.

### Ethics Approval

This study will be conducted in accordance with the Declaration of Helsinki. The ethics review board of the Amsterdam University Medical Center in the Netherlands approved this study (2022.0347, September 12, 2022). We will handle participant data in compliance with the General Data Protection Regulation (GDPR). All adolescents will give written informed consent, and legal guardians of adolescents aged 12-15 years will also provide consent before conducting any study procedures. All serious adverse events will be monitored and reported to the Medical Ethical Committee of the Amsterdam University Medical Center until the end of the study. In addition, during the measurements (directly, 3, 6, and 9 months after the BITT) we assess PTSD symptoms, comorbid symptoms, and risk behavior. In case of safety concerns, we contact the referring professional of the adolescent. Participants will receive a voucher of 10 euros (US $11.36) upon completing the final measurement.

## Results

This project was funded in June 2021 and approved by the institutional review board in September 2022. As of September 2022, we enrolled 104 participants. Data will be collected until December 2025. Results are expected to be published in the summer of 2026.

## Discussion

### Anticipated Findings

This study protocol describes the design of a multicenter, single-blinded RCT with the aim of examining the effectiveness of BITT compared to a WLCG in treating PTSD among adolescents. We hypothesize that the results will demonstrate a superior effect of BITT compared to WLCG and we expect a change in PTSD and comorbid symptoms among adolescents in the BITT group, while symptoms in adolescents in the WLCG will remain unchanged. We hypothesize that BITT can be a suitable treatment option for traumatized adolescents who do not receive the available treatment options due to high treatment avoidance, those who do not experience sufficient symptom decrease after regular trauma treatment or adolescents who drop out of treatment. For this group of severely traumatized adolescents, BITT could potentially be effective. Research in the adult population already has shown positive effects of BITT on PTSD symptoms [21]. However, not much research has been conducted among children and adolescents. Pilot studies show that BITT can be a safe (ie, no increased risk of suicidality) and effective treatment for adolescents with PTSD [25,26,30,31] but RCTs into its effectiveness are lacking and urgently needed.

There are several strengths to the proposed study. First, the implementation of a single-blinded RCT with a waitlist control group provides an opportunity to assess the effectiveness of the BITT and allow each participant to engage in the BITT. This is an important benefit since adolescents who participate in the BITT experience severe PTSD symptoms and comorbid symptoms and do not benefit sufficiently from regular treatment. Engaging in the BITT might offer an effective treatment for this severely traumatized group of adolescents. An RCT is considered the golden standard in clinical research, because, unlike other research models, it controls for selection bias, time effects, effects of administering questionnaires, and chance occurrences in potential treatment effects [62]. This enables us to research the true effects of a BITT on PTSD symptoms Notably, to the best of our knowledge, this RCT represents the first of its kind conducted across multiple (cross-cultural) sites, thereby broadening the scope and generalizability of the findings [63]. In addition, applying this study in multiple centers in the world might increase the quality of care worldwide because of the trainings and supervisions by the licensed TF-CBT and EMDR supervisors at the different sites and gives us the opportunity to treat traumatized adolescents across cultures [64].

Another strength of this study lies in the use of multiple variants of trauma-focused treatments combined with psychoeducation, psychomotor therapy, social sharing, and parental counseling. In this way, BITT incorporates several common elements from trauma-focused treatments for adolescents that are presumed to be effective [65]. Another novel element of the BITT is the therapist rotation, which seems to reduce the therapists’ fear of applying trauma treatment in adolescents with PTSD and therefore contributes to a better implementation of the treatment [44]. Notably, this study is innovative since there is no research yet about a BITT for adolescents that incorporates all these important elements.

In addition, in determining the effectiveness of the treatment program, we will use multiple sources of information which improve the reliability of the results [66]. We ask adolescents to report their symptoms over time, semistructured interviews are used which also incorporate clinical judgment, and caregivers are actively engaged as informants in monitoring treatment outcomes. In addition, we conduct measurements including a 3-, 6-, and 9-month follow-up, which enables us to detect an effect of BITT over time.

Despite these strengths, possible limitations need to be acknowledged. First, although an RCT design remains the golden standard in clinical research, it comes with challenges. RCTs often operate in controlled environments with strict eligibility criteria, which may limit the external validity or applicability to diverse populations. Furthermore, participants who volunteer for the study may differ systematically from those who do not, thus affecting the generalizability of the findings. However, by conducting the study across multiple (cross-cultural) sites, each with distinct teams serving diverse target populations, we aim to achieve a diverse and representative study sample, thereby minimizing the limitations to the fullest extent possible.

### Conclusions

This first, innovative study on the effectiveness of BITT may enhance treatment outcomes for PTSD in adolescents, by preventing dropout, reducing avoidance, shortening therapy duration, and empowering therapists by working together intensively. This research provides valuable insights across cultures for effective treatment of severely traumatized adolescents who do not benefit sufficiently from regular treatment.

## Abbreviations

BITT: Brief Intensive Trauma Treatment
CAPS-CA-5: Clinician-Administered PTSD Scale for *DSM-5*-Child/Adolescent Version
CATS-2: Child and Adolescent Trauma Screen 2
*DSM-5*: *Diagnostic and Statistical Manual of Mental Disorders (Fifth Edition)*
EMDR: eye movement desensitization and reprocessing
GDPR: General Data Protection Regulation
GEE: generalized estimating equation
LEC-5: Life Events Checklist for *DSM-5*
ORS: Outcome Rating Scale
PCL-5: PTSD Checklist for *DSM-5*
PMT: psychomotor therapy
PROMIS: Patient Reported Outcomes Measurement Information System
PTE: potentially traumatic event
PTSD: posttraumatic stress disorder
RCT: randomized controlled trial
SES: socioeconomic status
sPTSD: subclinical posttraumatic stress disorder
SRS: Session Rating Scale
TF-CBT: trauma-focused cognitive behavioral therapy
WLCG: waitlist control group

## Data Availability

Data sharing is not applicable to this article due to the sensitivity of the data.

## References

[1] Alisic E, Conroy R, Thoresen S, Forbes D, Bisson JL, Monson CM, Berliner L (2001). Epidemiology, clinical presentation, and developmental considerations in children and adolescents. Effective Treatments for PTSD: Practice Guidelines from the International Society for Traumatic Stress Studies. 3rd ed.

[2] Broekhof R, Nordahl HM, Bjørnelv Sigrid, Selvik SG (2022). Prevalence of adverse childhood experiences and their co-occurrence in a large population of adolescents: a Young HUNT 3 study. Soc Psychiatry Psychiatr Epidemiol.

[3] Carlson JS, Yohannan J, Darr CL, Turley MR, Larez NA, Perfect MM (2019). Prevalence of adverse childhood experiences in school-aged youth: A systematic review (1990–2015). International Journal of School & Educational Psychology.

[4] Alisic E, Zalta AK, van Wesel F, Larsen SE, Hafstad GS, Hassanpour K, Smid GE (2014). Rates of post-traumatic stress disorder in trauma-exposed children and adolescents: meta-analysis. Br J Psychiatry.

[5] (2013). Diagnostic and Statistical Manual of Mental Disorders 5th Edition.

[6] Ojeahere MI, Uwakwe R, Piwuna CG, Audu M, Goar SG, Armiyau A, Afolaranmi T (2021). Assessment of full and subsyndromal PTSD and quality of life of internally displaced older adults in northern Nigeria. Aging and Health Research.

[7] Zatzick DF, Jurkovich GJ, Fan MY, Grossman D, Russo J, Katon W, Rivara FP (2008). Association between posttraumatic stress and depressive symptoms and functional outcomes in adolescents followed up longitudinally after injury hospitalization. Arch Pediatr Adolesc Med.

[8] Lewis SJ, Arseneault L, Caspi A, Fisher HL, Matthews T, Moffitt TE, Odgers CL, Stahl D, Teng JY, Danese A (2019). The epidemiology of trauma and post-traumatic stress disorder in a representative cohort of young people in England and Wales. Lancet Psychiatry.

[9] Korte KJ, Allan NP, Gros DF, Acierno R (2016). Differential treatment response trajectories in individuals with subclinical and clinical PTSD. J Anxiety Disord.

[10] Hamblen JL, Norman SB, Sonis JH, Phelps AJ, Bisson JI, Nunes VD, Megnin-Viggars O, Forbes D, Riggs DS, Schnurr PP (2019). A guide to guidelines for the treatment of posttraumatic stress disorder in adults: An update. Psychotherapy (Chic).

[11] Thielemann JFB, Kasparik B, König J, Unterhitzenberger J, Rosner R (2022). A systematic review and meta-analysis of trauma-focused cognitive behavioral therapy for children and adolescents. Child Abuse Negl.

[12] John-Baptiste Bastien R, Jongsma HE, Kabadayi M, Billings J (2020). The effectiveness of psychological interventions for post-traumatic stress disorder in children, adolescents and young adults: a systematic review and meta-analysis. Psychol Med.

[13] (2018). Post-traumatic stress disorder. National Institute for Health and Care Excellence.

[14] Semmlinger V, Leithner C, Klöck Lea Maria, Ranftl L, Ehring T, Schreckenbach M (2024). Prevalence and Predictors of Nonresponse to Psychological Treatment for PTSD: A Meta-Analysis. Depress Anxiety.

[15] Diehle J, Opmeer BC, Boer F, Mannarino AP, Lindauer RJL (2015). Trauma-focused cognitive behavioral therapy or eye movement desensitization and reprocessing: what works in children with posttraumatic stress symptoms? A randomized controlled trial. Eur Child Adolesc Psychiatry.

[16] Jensen TK, Braathu N, Birkeland MS, Ormhaug SM, Skar AS (2022). Complex PTSD and treatment outcomes in TF-CBT for youth: a naturalistic study. Eur J Psychotraumatol.

[17] Ormhaug SM, Jensen TK (2018). Investigating treatment characteristics and first-session relationship variables as predictors of dropout in the treatment of traumatized youth. Psychother Res.

[18] Simmons C, Meiser-Stedman R, Baily H, Beazley P (2021). A meta-analysis of dropout from evidence-based psychological treatment for post-traumatic stress disorder (PTSD) in children and young people. Eur J Psychotraumatol.

[19] Dittmann I, Jensen TK (2014). Giving a voice to traumatized youth-experiences with Trauma-Focused Cognitive Behavioral Therapy. Child Abuse Negl.

[20] Imel ZE, Laska K, Jakupcak M, Simpson TL (2013). Meta-analysis of dropout in treatments for posttraumatic stress disorder. J Consult Clin Psychol.

[21] Hoppen TH, Kip A, Morina N (2023). Are psychological interventions for adult PTSD more efficacious and acceptable when treatment is delivered in higher frequency? A meta-analysis of randomized controlled trials. J Anxiety Disord.

[22] Oprel DAC, Hoeboer CM, Schoorl M, de Kleine RA, Cloitre M, Wigard IG, van Minnen A, van der Does W (2021). Effect of Prolonged Exposure, intensified Prolonged Exposure and STAIR+Prolonged Exposure in patients with PTSD related to childhood abuse: a randomized controlled trial. Eur J Psychotraumatol.

[23] Sciarrino NA, Warnecke AJ, Teng EJ (2020). A Systematic Review of Intensive Empirically Supported Treatments for Posttraumatic Stress Disorder. J Trauma Stress.

[24] Matthijssen SJMA, Menses SDF, Huisman-van Dijk HM (2024). The effects of an intensive outpatient treatment for PTSD. Eur J Psychotraumatol.

[25] Hendriks L, de Kleine RA, Heyvaert M, Becker ES, Hendriks GJ, van Minnen A (2017). Intensive prolonged exposure treatment for adolescent complex posttraumatic stress disorder: a single-trial design. J Child Psychol Psychiatry.

[26] van Pelt Y, Fokkema P, de Roos C, de Jongh A (2021). Effectiveness of an intensive treatment programme combining prolonged exposure and EMDR therapy for adolescents suffering from severe post-traumatic stress disorder. Eur J Psychotraumatol.

[27] van Ee E, de Beijer D, Florisson D, Geuskens F (2024). Making sense of change after Intensive Trauma Treatment: a mixed-methods study into adolescents' experience of efficacy. Child Adolesc Psychiatry Ment Health.

[28] Tijsseling I, Noordende ATV, Zijlstra BJH, Merbis M, Veen SCV (2024). The effectiveness and tolerability of an intensive outpatient trauma treatment program for adolescents with PTSD. J EMDR Prac Res.

[29] Rentinck EM, van Mourik R, de Jongh A, Matthijssen SJMA (2025). Effectiveness of an intensive outpatient treatment programme combining prolonged exposure and EMDR therapy for adolescents and young adults with PTSD in a naturalistic setting. Eur J Psychotraumatol.

[30] Cabrera N, Moffitt G, Jairam R, Barton G (2020). An intensive form of trauma focused cognitive behaviour therapy in an acute adolescent inpatient unit: An uncontrolled open trial. Clin Child Psychol Psychiatry.

[31] Ooms-Evers M, van der Graaf-Loman S, van Duijvenbode N, Mevissen L, Didden R (2021). Intensive clinical trauma treatment for children and adolescents with mild intellectual disability or borderline intellectual functioning: A pilot study. Res Dev Disabil.

[32] Roque-Lopez S, Llanez-Anaya E, Álvarez-López María Jesús, Everts M, Fernández Daniel, Davidson RJ, Kaliman P (2021). Mental health benefits of a 1-week intensive multimodal group program for adolescents with multiple adverse childhood experiences. Child Abuse Negl.

[33] Reeson M, Polzin W, Pazderka H, Agyapong V, Greenshaw AJ, Hnatko G, Wei Y, Szymanski L, Silverstone PH (2020). A Novel 2-week Intensive Multimodal Treatment Program for Child Sexual Abuse (CSA) Survivors is Associated with Mental Health Benefits for Females aged 13-16. J Can Acad Child Adolesc Psychiatry.

[34] Albisser N, Westerveld M, Kooij L, de Keizer-Altink M, Lindauer R (2024). Korte intensieve traumabehandeling bij jongeren. Kind Adolesc Prakt.

[35] van Meijel E, Ensink J, Verlinden E, Lindauer R (2019). Klinisch interview voor PTSS bij kinderen en adolescenten. Houten: BSL.

[36] Nader K, Kriegler KA, Blake DD, Pynoos RS, Newman E, Weathers FW (1996). Clinician-Administered PTSD Scale For Children and Adolescents (CAPS-CA). APA PsycNet.

[37] Team RC (2000). R language definition. Vienna, Austria: R foundation for statistical computing.

[38] (2021). Castor electronic data capture. Castor.

[39] Gutermann J, Schreiber F, Matulis S, Schwartzkopff L, Deppe J, Steil R (2016). Psychological Treatments for Symptoms of Posttraumatic Stress Disorder in Children, Adolescents, and Young Adults: A Meta-Analysis. Clin Child Fam Psychol Rev.

[40] van den Hout MA, Engelhard IM (2012). How does EMDR work?. J Exp Psychopathol.

[41] de Roos C, van der Oord S, Zijlstra B, Lucassen S, Perrin S, Emmelkamp P, de Jongh A (2017). Comparison of eye movement desensitization and reprocessing therapy, cognitive behavioral writing therapy, and wait-list in pediatric posttraumatic stress disorder following single-incident trauma: a multicenter randomized clinical trial. J Child Psychol Psychiatry.

[42] Shapiro F (2017). Eye Movement Desensitization and Reprocessing (EMDR) Therapy: Basic Principles, Protocols, and procedures.

[43] Van Minnen A, Voorendonk EM, Rozendaal L, de Jongh A (2020). Sequence matters: Combining Prolonged Exposure and EMDR therapy for PTSD. Psychiatry Res.

[44] Van Minnen A, Hendriks L, Kleine RD, Hendriks GJ, Verhagen M, De Jongh A (2018). Therapist rotation: a novel approach for implementation of trauma-focused treatment in post-traumatic stress disorder. Eur J Psychotraumatol.

[45] Rosenbaum S, Vancampfort D, Steel Z, Newby J, Ward PB, Stubbs B (2015). Physical activity in the treatment of Post-traumatic stress disorder: A systematic review and meta-analysis. Psychiatry Res.

[46] van de Kamp MM, Scheffers M, Emck C, Fokker TJ, Hatzmann J, Cuijpers P, Beek PJ (2023). Body-and movement-oriented interventions for posttraumatic stress disorder: An updated systematic review and meta-analysis. J Trauma Stress.

[47] Kooij L, Lindauer Rjl (2017). Child and Adolescent Trauma Screen. Nederlands Jeugdinstutuut.

[48] Sachser C, Berliner L, Holt T, Jensen TK, Jungbluth N, Risch E, Rosner R, Goldbeck L (2017). International development and psychometric properties of the Child and Adolescent Trauma Screen (CATS). J Affect Disord.

[49] Diehle Julia, de Roos Carlijn, Boer Frits, Lindauer RJL (2013). A cross-cultural validation of the Clinician Administered PTSD Scale for Children and Adolescents in a Dutch population. Eur J Psychotraumatol.

[50] Terwee CB, Roorda LD, de Vet HCW, Dekker J, Westhovens R, van Leeuwen J, Cella D, Correia H, Arnold B, Perez B, Boers M (2014). Dutch-Flemish translation of 17 item banks from the patient-reported outcomes measurement information system (PROMIS). Qual Life Res.

[51] van Muilekom MM, Luijten MAJ, van Litsenburg RRL, Grootenhuis MA, Terwee CB, Haverman L (2021). Psychometric properties of the Patient-Reported Outcomes Measurement Information System (PROMIS®) Pediatric Anger Scale in the Dutch general population. Psychol Assess.

[52] Klaufus LH, Luijten MAJ, Verlinden E, van der Wal MF, Haverman L, Cuijpers P, Chinapaw MJM, Terwee CB (2021). Psychometric properties of the Dutch-Flemish PROMIS pediatric item banks Anxiety and Depressive Symptoms in a general population. Qual Life Res.

[53] (2009). EQ-5D-3L. EuroQol Group.

[54] Golicki D, Młyńczak K (2022). Measurement Properties of the EQ-5D-Y: A Systematic Review. Value Health.

[55] Weathers FW, Litz BT, Keane TM, Palmieri PA, Marx BP, Schnurr PP (2013). The PTSD checklist for DSM-5 (PCL-5). National Center for PTSD.

[56] Forkus SR, Raudales AM, Rafiuddin HS, Weiss NH, Messman BA, Contractor AA (2023). The Posttraumatic Stress Disorder (PTSD) Checklist for DSM-5: A Systematic Review of Existing Psychometric Evidence. Clin Psychol (New York).

[57] Weathers FW, Blake DD, Schnurr PP, Kaloupek DG, Marx BP, Keane TM (2013). The life events checklist for DSM-5 (LEC-5). National Center for PTSD.

[58] Gray MJ, Litz BT, Hsu JL, Lombardo TW (2004). Psychometric properties of the life events checklist. Assessment.

[59] Miller SD, Duncan BL, Brown J, Sparks JA, Claud DA (2003). The outcome rating scale: a preliminary study of the reliability, validity, and feasibility of a brief visual analog measure. Journal of brief Therapy.

[60] Duncan B, Miller S, Sparks J, Claud DA (2003). The session rating scale: preliminary psychometric properties of a "working "alliance measure. J Brief Ther.

[61] Campbell A, Hemsley S (2009). Outcome Rating Scale and Session Rating Scale in psychological practice: Clinical utility of ultra-brief measures. Clinical Psychologist.

[62] Edwards P (2010). Questionnaires in clinical trials: guidelines for optimal design and administration. Trials.

[63] Sprague S, Matta JM, Bhandari M, Dodgin David, Clark Charles R, Kregor Phil, Bradley Gary, Little Lester, Anterior Total Hip Arthroplasty Collaborative (ATHAC) Investigators (2009). Multicenter collaboration in observational research: improving generalizability and efficiency. J Bone Joint Surg Am.

[64] Draguns JG (2013). Cross-cultural and international extensions of evidence-based psychotherapy: toward more effective and sensitive psychological services everywhere. Psychologia.

[65] Kooij LH, van der Pol T M, Daams JG, Hein IM, Lindauer RJL (2022). Common elements of evidence-based trauma therapy for children and adolescents. Eur J Psychotraumatol.

[66] Maruyama G, Ryan C (2014). Research Methods in Social Relations.

